# Ceria/cobalt borate hybrids as efficient electrocatalysts for water oxidation under neutral conditions†

**DOI:** 10.1039/c9na00356h

**Published:** 2019-07-19

**Authors:** Xuemei Zhou, Sijia Guo, Qiran Cai, Shaoming Huang

**Affiliations:** School of Material and Energy, Guangzhou Key Laboratory of Low Dimensional Materials and Energy Devices, Guangdong University of Technology Guangzhou 51006 P. R. China smhuang@gdut.edu.cn; Institute for Frontier Materials, Deakin University, Geelong Waurn Ponds Campus Victoria 3216 Australia

## Abstract

Oxygen evolution reaction (OER) catalysts are of importance for electrochemical water splitting and fuel generation. Despite enormous efforts, the design and development of OER catalysts with high catalytic activities under neutral conditions are highly desired but still remain a great challenge. Herein, we report a room temperature chemical route to prepare *x*ceria/cobalt borate (*x*CeO_2_/Co–Bi) hybrids as efficient OER catalysts by tuning the molar ratio of Ce/Co (*x* represents the amount of CeO_2_). The optimised catalyst (20CeO_2_/Co–Bi hybrid) was found to exhibit remarkable OER catalytic activity with an overpotential of 453 mV at a current density of 10 mA cm^−2^, Tafel slope of 120 mV dec^−1^ and long-term stability in neutral medium due to its good conductivity, mass transportation and strong synergetic coupling effects, showing the potential of Co-based electrochemical materials for practical application in energy storage devices.

## Abstract 2

Oxygen evolution reaction (OER) catalysts are of importance for electrochemical water splitting and fuel generation.

## Graphical abstract

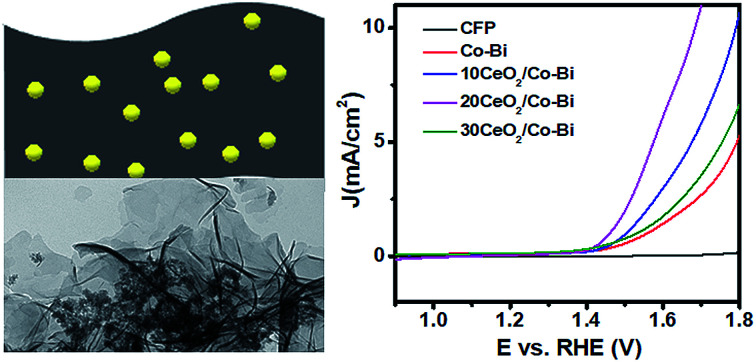

## 1. Introduction

Many efforts have been made to explore alternative energy storage and conversion systems to solve the increasing environmental problems and energy crisis.^1–3^ Hydrogen has been considered as a clean alternative energy due to its high energy output and carbon-neutral combustion products,^2,4^ and electrochemical water splitting is thought to be a promising technology to convert electrical energy into chemical fuels for hydrogen storage.^5–7^ However, the oxygen evolution reaction (OER) significantly affects the overall water-splitting efficiency due to its sluggish kinetics with an energy-intensive reaction and high overpotential.^8–11^ Although many electrocatalysts have been explored to achieve high current density at low overpotentials and expedite the OER reaction kinetics process,^8,9,12,13^ noble metal oxides (*e.g.* RuO_2_ or IrO_2_), operating in acidic or alkaline solutions, are the most active OER electrocatalysts. However, their high cost, scarcity, and the requirement for extreme pH conditions have greatly hindered the large-scale application of these OER catalysts.^12,14–16^ Therefore, it is highly desirable to develop effective non-noble metal OER catalytic alternatives to improve the efficiency of water oxidation in a near-neutral environment.

Oxygen-evolving complexes composed of manganese and calcium are well known as natural water-splitting complexes.^17,18^ Inspired by this thought, a new category of artificial water oxidation electrocatalysts with amorphous features consisting of metals (such as nickel and cobalt), oxygen, and inorganic borate, namely nickel–borate (Ni–Bi) or cobalt–borate (Co–Bi), has attracted significant attention due to their low cost, high intrinsic activity, and superior stability.^13,14,19–22^ For example, Nocera *et al.* reported that a nickel–borate (Ni–Bi) film, electro-deposited on indium tin oxide (ITO), had high catalytic activity towards electrochemical water oxidation under near-neutral conditions, achieving a current density of 1 mA cm^−2^ in 0.1 M borate buffer at an overpotential of 425 mV.^19^ A nickel–borate nanoarray supported by carbon cloth (Ni–Bi/CC) yielded a geometrical catalytic current density of 10 mA cm^−2^ at an overpotential of 470 mV.^14^ Recently, amorphous Co–Bi ultrathin nanosheets were designed for electrochemical water oxidation under neutral conditions.^13,23^ However, the application of Co–Bi as a catalyst is limited by its poor OER kinetics and mass transfer ability, which can be solved by adding OER co-catalysts.

As one of the most important rare earth oxides, ceria (CeO_2_) has been usually used in the electrochemical field due to the following advantages:^7,24–27^ (i) flexible transition between the Ce^3+^ and Ce^4+^ oxidation states, which can provide the opportunity to generate strong electronic interactions with other matrices and therefore probably improves the catalytic performance; (ii) good electronic/ionic conductivity, which can improve charge transfer and promote the reaction process; (iii) reversible surface oxygen ion exchange, which will absorb O_2_ produced during the OER and enhance the OER activity; (iv) large oxygen-storage capacity, which is helpful to form and repair oxygen vacancies on the CeO_2_ surface, binding adsorbates much more strongly than normal oxide sites.^24^ Therefore, the introduction of CeO_2_ onto Co–Bi nanosheets will be beneficial for the formation of hydroperoxy species (OOH_ad_) on the surface of the hybrids owing to the high mobility of oxygen vacancies. Furthermore, the chemical synergistic effect between Co–Bi nanosheets and CeO_2_ would also improve the OER catalytic performance. Thus, the CeO_2_/Co–Bi hybrids should be efficient candidates to improve the electrocatalytic activity for OER in neutral medium.

In this work, we report a room temperature chemical route to prepare *x*CeO_2_/Co–Bi hybrids (10CeO_2_/Co–Bi, 20CeO_2_/Co–Bi, 30CeO_2_/Co–Bi; *x* represents the amount of CeO_2_) through tuning the molar ratio of Ce/Co, and their OER catalytic activities were compared. It was found that 20CeO_2_/Co–Bi exhibited the highest OER catalytic activity with an overpotential of 453 mV at a current density of 10 mA cm^−2^, Tafel slope of around 120 mV dec^−1^ and long-term stability in phosphate-buffered saline (PBS) solution because of the good conductivity, mass transportation and strong synergetic coupling effects.

## 2. Experimental

All chemicals were commercially available and used without further purification. Cobalt nitrate hexahydrate (Co(NO_3_)_2_·6H_2_O), cerium nitrate hexahydrate (Ce(NO_3_)_3_·6H_2_O) and sodium borohydride (NaBH_4_) were purchased from Sigma. Milli-Q water (18.2 MΩ cm) was used in this work.

### 2.1 Synthesis of CeO_2_/Co–Bi composites

**Fig. 1:**
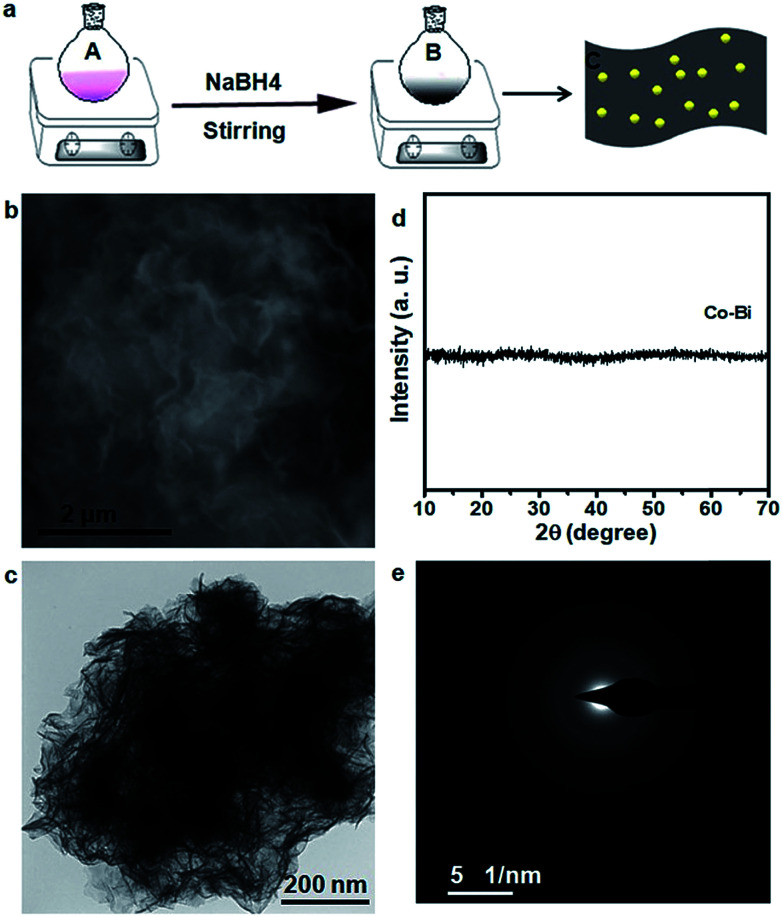
(a) Schematic illustration of the synthesis of the CeO_2_/Co–Bi hybrids by a room temperature chemical method. A refers to the solution consisting of Co(NO_3_)_2_·6H_2_O and Ce(NO_3_)_3_·6H_2_O. B refers to the suspended products of CeO_2_/Co–Bi. C refers to the structure of CeO_2_/Co–Bi hybrids. (b) SEM and (c) TEM images, (d) XRD patterns, and (e) SAED pattern of pure Co–Bi nanosheets.

The CeO_2_/Co–Bi samples were prepared by a room temperature chemical synthesis method as illustrated in Fig. 1a. In particular, *n* mmol (*n* = 0.1, 0.2 and 0.3) Ce(NO_3_)_3_·6H_2_O and (1 − *n*) mmol Co(NO_3_)_2_·6H_2_O were added into a flask with 100 ml deionized water under continuous and vigorous stirring for complete dissolution (marked as A). Then, 5 mL NaBH_4_ solution (0.5 mol L^−1^) was added to A, and the mixed solution was kept at room temperature for 40 min under strong stirring; the solution gradually turned into black suspended solids (marked as B). The suspended products were collected by centrifugation and laved with deionized water and ethanol several times. The as-prepared products were finally dried in a vacuum overnight for further characterization. Co–Bi hierarchical nanosheets were prepared by the same procedure but without using Ce(NO_3_)_3_·6H_2_O.

### 2.2 Characterization

The surface area was measured by nitrogen physisorption (Micromeritics, ASAP 2020 HD88) based on the Brunauer–Emmett–Teller (BET) method. The phase evolution of the as-synthesized nanostructures was monitored by powder X-ray diffraction (XRD). The XRD patterns with diffraction intensity *versus* 2*θ* were recorded with a Shimadzu X-ray diffractometer (Model 6000) using Cu Kα radiation. X-ray photoelectron spectra (XPS) were acquired on a Thermo Scientific Model K-Alpha with Al Kα as the excitation source. Raman spectra were recorded at room temperature using an XY Dilor spectrograph equipped with a Spex CCD detector (2000 × 800 pixels). Spectra were recorded in microconfiguration with a laser impact of *ca.* 1 μm diameter. The excitation source was the 633 nm line of an argon laser at the power level of 5.4 mW. Scanning electron microscopy (SEM) was performed on a Hitachi Su8010 scanning electron microscope. Transmission electron microscopy (TEM) studies were conducted on a Hitachi HT-7700 transmission electron microscope with an accelerating voltage of 120 kV. High-resolution TEM, selected area electron diffraction (SAED), electron energy loss spectroscopy (EELS), and energy-dispersive X-ray spectroscopy (EDS) were conducted on a JEOL JEM 2100F transmission electron microscope with an accelerating voltage of 200 kV.

### 2.3 Electrochemical measurements

Electrocatalytic activities assessed by linear sweep voltammetry (LSV) and chronoamperometry were measured on a CHI 660D electrochemical workstation (CH Instruments, Shanghai, China) with a standard three-electrode system in 0.1 M PBS aqueous solution (pH = 7.4). The counter and reference electrodes were platinum wire and standard Ag/AgCl (3 M KCl), respectively. The catalyst with a loading of 0.56 mg cm^−2^ on carbon fiber paper (CFP) was used as the working electrode. The scanning rate for LSV measurements was 5 mV s^−1^. Rotating-disk electrode voltammograms were obtained at a scan rate of 10 mV s^−1^. Electrochemical impedance spectroscopy (EIS) was performed on the AUTOLAB PGSTAT204 electrochemical workstation in the frequency range from 0.01 Hz to 100 kHz at an open circuit potential, with 10 mV as the amplitude potential.

## 3. Results and discussion

**Fig. 2:**
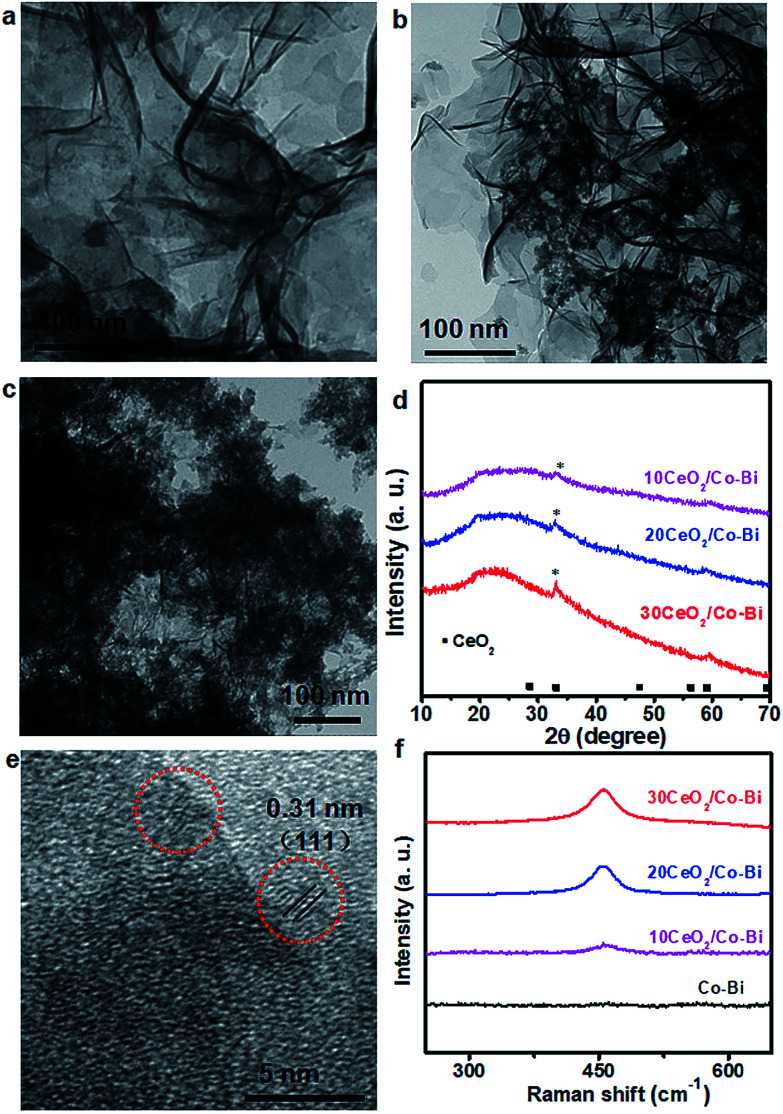
Characterization of CeO_2_/Co–Bi composites. TEM images (a–c) and (d) XRD patterns of 10CeO_2_/Co–Bi, 20CeO_2_/Co–Bi and 30CeO_2_/Co–Bi, respectively. (e) HRTEM images of 20CeO_2_/Co–Bi and (f) Raman spectra for Co–Bi, 10CeO_2_/Co–Bi, 20CeO_2_/Co–Bi and 30CeO_2_/Co–Bi.

The morphology and composition of the as-prepared products were characterized by SEM, TEM and XRD, as shown in Fig. 1. The SEM (Fig. 1b) and TEM (Fig. 1c) images of the as-prepared Co–Bi nanosheets show a hierarchical structure comprising aggregated ultrathin nanosheets. XRD patterns (Fig. 1c) show that Co–Bi ultrathin nanosheets have no diffraction peaks, implying the presence of amorphous structures, which is in agreement with a previous report.^13^ The amorphous structure was further confirmed by selected area electron diffraction (SAED) as shown in Fig. 1e. Some new nanoparticles were observed to be uniformly coated on the surface of Co–Bi nanosheets after introducing the source of Ce (Fig. 2a–c). In addition, the amount of the particles increases with the incremental amount of Ce source, but excess particles would destroy the structure of the Co–Bi nanosheets (Fig. 2a–c.). A new diffraction peak at 33° (Fig. 2d, marked by *), indexed to the (200) of CeO_2_,^24,26^ was detected from CeO_2_/Co–Bi composites compared to pure Co–Bi nanosheets, and its intensity increased with the increase in the Ce source (Fig. 1d). No more XRD peaks of CeO_2_ (■ represents the peaks of pure CeO_2_, JCPDS no. 34-0394) were detected due to the small size of CeO_2_ particles (<5 nm), their good dispersibility on the nanosheets and the effect of the amorphous structure of Co–Bi nanosheets. Additionally, EDS mapping and EELS spectra show the presence and the homogeneous distribution of the elements Co, B, O, and Ce in the as-prepared 20CeO_2_/Co–Bi composite, as shown in Fig. S1 and S2† which depicts the K-edge of B at 188 eV,^23,28^ L-edges of Co at 780 eV and M-edges of Ce at 890 eV.^29–31^ In order to further analyse the compositions, we chose 20CeO_2_/Co–Bi as a representative to be studied by HRTEM (Fig. 2e). The nanoparticles on the surface of the Co–Bi nanosheets show a lattice fringe with a distance of 0.31 nm, which was assigned to (111) of CeO_2_. The results demonstrate that the CeO_2_/Co–Bi composites have been successfully prepared.

Raman spectroscopy was applied to further confirm the presence of CeO_2_ in the as-prepared composites because the F_2g_ mode of CeO_2_ of the fluorite structure is Raman active at 465 cm^−1^.^32,33^Fig. 2f shows the Raman spectra collected from the composites (black line indicates pure Co–Bi nanosheets), and the intensity increases with the increase in the amount of CeO_2_. The ∼10 cm^−1^ shift of Raman frequency between our samples and literature reports^33–35^ is attributed to the size effects, which is consistent with a previous report.^36^

The specific surface area of the as-grown samples was measured from the standard BET procedure (Fig. S3†). The BET surface areas of 10CeO_2_/Co–Bi, 20CeO_2_/Co–Bi and 30CeO_2_/Co–Bi were 249.8 m^2^ g^−1^, 290.2 m^2^ g^−1^ and 330.1 m^2^ g^−1^, respectively, much higher than that of pure Co–Bi nanosheets (134.0 m^2^ g^−1^). It is generally accepted that a larger surface area means better catalytic activity.

**Fig. 3:**
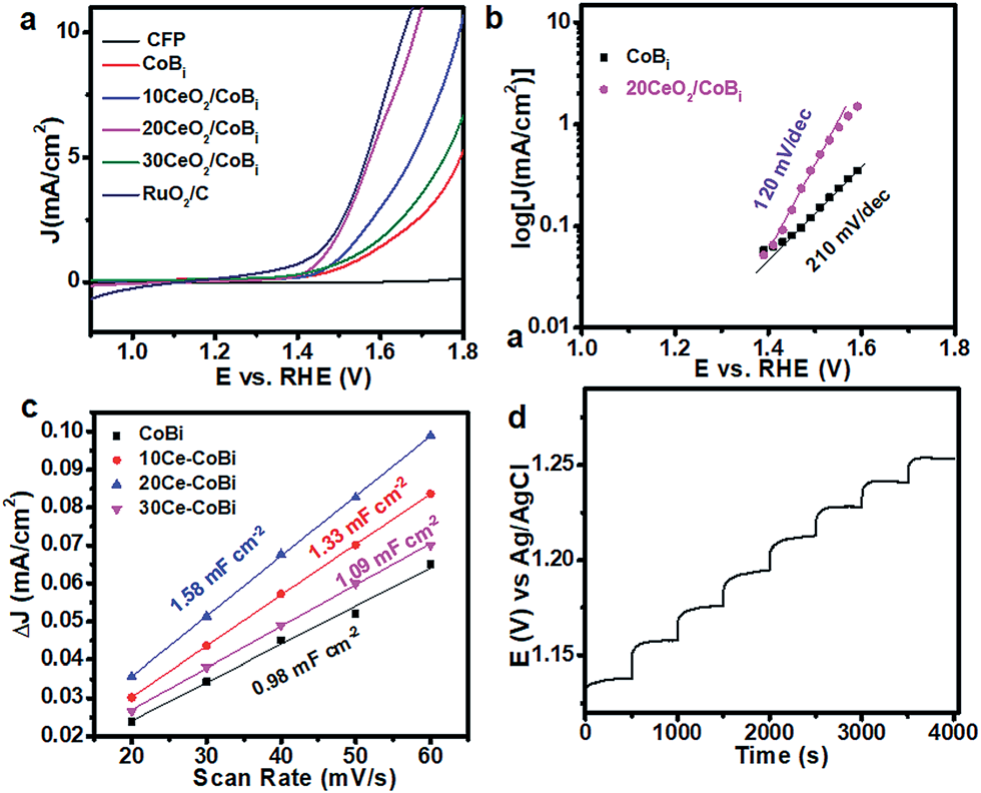
(a) Linear sweep voltammograms of all catalytic (CFP, Co–Bi, 10CeO_2_/Co–Bi, 20CeO_2_/Co–Bi, 30CeO_2_/Co–Bi and commercial RuO_2_/C) electrodes in 0.1 M PBS without *iR* correction; (b) Tafel slope for Co–Bi and 20CeO_2_/Co–Bi; (c) multi-current process of 20CeO_2_/Co–Bi in 0.1 M PBS. The current density started at 2 mA cm^−2^ and ended at 9 mA cm^−2^, with an increment of about 1 mA cm^−2^ per 500 s, without *iR* correction and (d) charging current density differences plotted *versus* scan rate. The linear slope, equivalent to twice the double-layer capacitance *C*_dl_, was used to represent the ECSA.

To evaluate the OER activities of the CeO_2_/Co–Bi composites, the catalysts were loaded onto the CFP electrode with a density of 0.56 mg cm^−2^ by drop casting. The potentials reported in this work were calibrated to the RHE using the following equation: *E*(RHE) = *E*(Ag/AgCl) + (0.197 + 0.0591 × pH) V.^13^Fig. 3a shows the LSV curves of a bare CFP substrate, Co–Bi, 10CeO_2_/Co–Bi, 20CeO_2_/Co–Bi, 30CeO_2_/Co–Bi and commercial RuO_2_/C electrodes in 0.1 M PBS. Compared to the strong catalytic current from electrodes with catalysts, almost no current density from the bare CFP substrate was detected at 1.8 V (*vs.* RHE), indicating that CFP is inactive towards O_2_ evolution and a background correction for the CFP support is unnecessary for all catalytic electrodes. The overpotentials at the specified current density of 5 mA cm^−2^, the current densities at 1.8 V (*vs.* RHE) and the Tafel slopes of all catalysts, obtained in 0.1 M PBS, are summarized in Table 1. The 20CeO_2_/Co–Bi electrode demands an overpotential of only 346 mV to deliver a catalytic current density of 5 mA cm^−2^, much lower than those of 10CeO_2_/Co–Bi (446 mV) and 30CeO_2_/Co–Bi (525 mV) under the same catalytic current density, respectively. For comparison, the electrocatalytic OER activity of Co–Bi nanosheets was measured as well, obtaining an overpotential of 560 mV at a current density of 5 mA cm^−2^, much higher than those of CeO_2_/Co–Bi composite electrodes. In addition, we obtained an overpotential of 453 mV from the 20CeO_2_/Co–Bi electrode at a current density of 10 mA cm^−2^, which is close to that of RuO_2_/C (430 mV) and also comparable to the values reported previously under neutral conditions (Table S1†).^13,14,23,37–44^ The current density of the 20CeO_2_/Co–Bi electrode at 1.8 V (*vs.* RHE) was 19.2 mA cm^−2^, not only much larger than 11.4, 7.2, and 5.7 mA cm^−2^ obtained from 10CeO_2_/Co–Bi, 30CeO_2_/Co–Bi and Co–Bi electrodes, respectively, but also superior to those of A-CoS_4.6_O_0.6_ PNCs (4.59 mA cm^−2^) and Co–Bi NS/G (14.4 mA cm^−2^) measured at the same potential (1.8 V *vs.* RHE).^13,45^ All these results indicate the high OER catalytic activity of the 20CeO_2_/Co–Bi hybrid under neutral conditions.

**Table 1:** Summary of the OER electrocatalytic activities of the samples in phosphate-buffered saline solution (pH = 7.4)

Sample	*η* a@5 (mA cm^−2^)	*J* b@1.8 V (*vs.* RHE)	Tafel slope (mV dec^−1^)	BET surface area (m^2^ g^−1^)	Capacitance (mF cm^−2^)
Co–Bi	560	5.7	210	134.0	0.98
10CeO_2_/Co–Bi	446	11.4	160	249.8	1.33
20CeO_2_/Co–Bi	346	19.2	120	290.2	1.58
30CeO_2_/Co–Bi	525	7.2	198	330.1	1.09

a *η* refers to the overpotential (mV) of the samples at a current density of 5 mA cm^−2^.

b *J* refers to the current densities of the catalysts at a specific overpotential.

We also fitted the polarization curves (Fig. 3a) using the Tafel equation *η* = *b* log(*j*/*j*_0_), where *η* represents the overpotential, *b* is the Tafel slope, *j* refers to the current density and *j*_0_ is the exchange current density.^9^ Accordingly, the derived Tafel slope of 120 mV dec^−1^ from 20CeO_2_/Co–Bi was much lower than the slope of 210 mV dec^−1^ from Co–Bi (Fig. 3b) and close to or even lower than the values measured from other reported catalysts, including 3D Ni–Bi nanowire arrays (107 mV dec^−1^),^14^ Co–Bi/graphene composites (160 mV dec^−1^),^13^ Ni–Bi/RGO (176 mV dec^−1^)^46^ and 3D Co–Pi nanowires (187 mV dec^−1^),^12^ suggesting the high OER activity of 20CeO_2_/Co–Bi. Additionally, Fig. 3c shows a multi-step chronopotentiometric curve for the 20CeO_2_/Co–Bi composite electrode at current densities from 2 to 9 mA cm^−2^ (*ca.* 1 mA cm^−2^ per 500 s). The potential immediately levels off at 1.13 V (*vs.* Ag/AgCl) at the initial current value and remains unchanged for 500 s, and the other steps also show similar results, implying good conductivity, mass transportation, and mechanical robustness of this electrode.^47,48^ These results further demonstrate that 20CeO_2_/Co–Bi composite electrodes have excellent OER catalytic performance.

The reasons for the enhancement of electrochemical catalytic OER activity of CeO_2_/Co–Bi compared to that of Co–Bi are summarised as follows. First, CeO_2_/Co–Bi composites have a much larger surface area than Co–Bi and thus more catalytically active sites, which can significantly improve the OER process (Table 1). Although the surface area of the 30CeO_2_/Co–Bi composite (330.1 m^2^ g^−1^) is larger than that of the 20CeO_2_/Co–Bi composite (290.2 m^2^ g^−1^), 30CeO_2_/Co–Bi shows worse OER performance than 20CeO_2_/Co–Bi because the nanosheet structure of 30CeO_2_/Co–Bi has been destroyed (Fig. 2c). Electrochemical impedance spectroscopy (EIS) was used to further investigate the detailed characteristics of Co–Bi, 10CeO_2_/Co–Bi, 20CeO_2_/Co–Bi, and 30CeO_2_/Co–Bi as capacitive electrodes (see ESI Fig. S4†). The semicircular characteristic of EIS curves suggests the smallest charge transfer and the best charge conductivity of 20CeO_2_/Co–Bi compared to those of other catalysts (Co–Bi, 10CeO_2_/Co–Bi and 30CeO_2_/Co–Bi) and thus the highest OER catalytic activity among the samples.

Second, CeO_2_/Co–Bi composites have a larger electrochemical surface area (ECSA) compared to Co–Bi. A larger ECSA means more accessible surface permeation and thus higher catalytic activity. According to the CV curves of Co–Bi, 10CeO_2_/Co–Bi, 20CeO_2_/Co–Bi, and 30CeO_2_/Co–Bi (see ESI Fig. S5†), capacitive currents as a function of the scan rate of the samples are plotted in Fig. 3c. The double-layer capacitance (*C*_dl_) of each sample was estimated from the slope of the corresponding linear fitting, and therefore, one can obtain the value of ECSA, which is double the *C*_dl_ value.^45^ Obviously, 20CeO_2_/Co–Bi has the highest *C*_dl_ among the four samples (Fig. 3d), and thus, the largest ECSA contributes to the best catalytic performance.

**Fig. 4:**
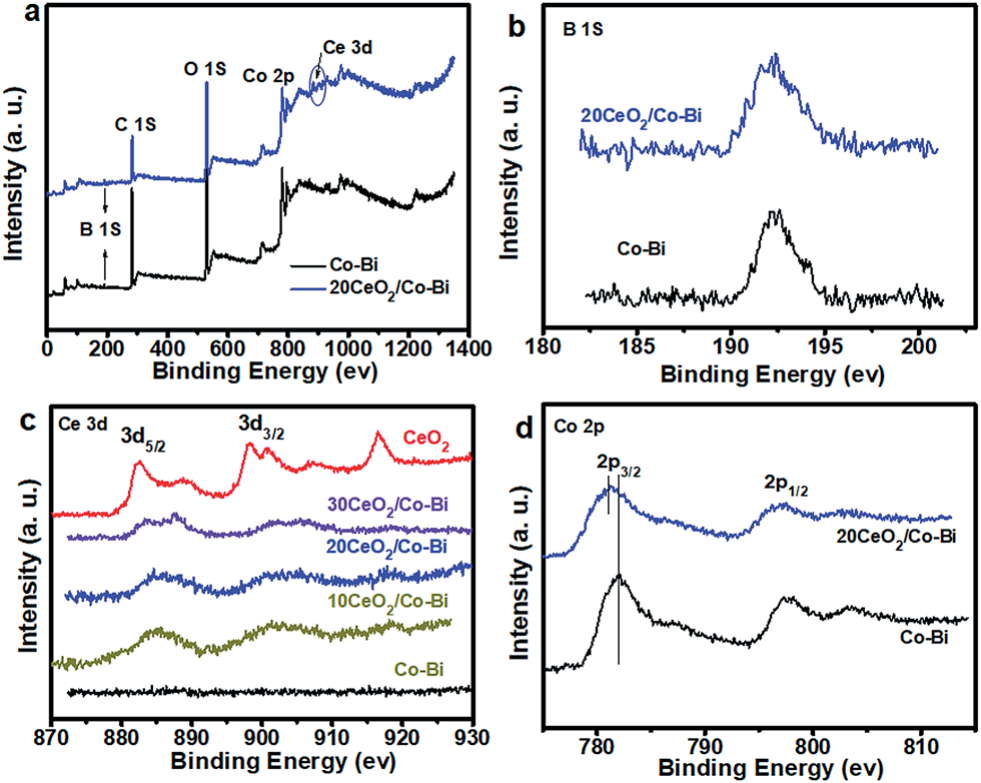
XPS spectra of Co–Bi and 20CeO_2_/Co–Bi composites (a), and high-resolution XPS spectra for B (b), Ce (c) and Co (d).

Lastly, in order to investigate the strong electronic interactions between Co–Bi and CeO_2_, XPS was applied to characterize pure CeO_2_, Co–Bi, 20CeO_2_/Co–Bi, 20CeO_2_/Co–Bi and 30CeO_2_/Co–Bi hybrids, and the results are plotted in Figs 4, S6 and S7.† All the spectra were referenced to the aliphatic carbon at a binding energy (BE) of 284.5 eV. The survey spectra of 20CeO_2_/Co–Bi showed the presence of Co, B, O and Ce, but no Ce was detected from Co–Bi nanosheets (Fig. 4c), consistent with EDS and EELS results (see ESI Fig. S1 and S2†).

As shown in Fig. 4b, the peak at 192.5 eV is assigned to the core level of B^3+^ in borate species. The O 1s spectra of pure CeO_2_, Co–Bi, 10CeO_2_/Co–Bi, 20CeO_2_/Co–Bi and 20CeO_2_/Co–Bi are shown in Fig. S8.† The peak at 529.4 eV is ascribed to the O 1s of Ce–O and that at 531.9 eV corresponds to O 1s of the central oxygen atoms in borate.^25,49^ For high-resolution Ce 3d spectra of pure CeO_2_ (Fig. 4c), the peaks located at 896–926 eV correspond to Ce 3d_3/2_, and the peaks located at 880–892 eV are consistent with Ce 3d_5/2_, which demonstrated the coexistence of Ce^3+^ and Ce^4+^ in CeO_2_. However, after introducing CeO_2_ NPs onto the Co–Bi nanosheets, the ratio of Ce^3+^ : Ce^4+^ in the CeO_2_/Co–Bi hybrid changed compared with that of pure CeO_2_, suggesting that the valence states of Ce in the CeO_2_/Co–Bi hybrid have rearranged.^25,26^ As shown in Fig. 4d, the binding energy of Co 2p from 20CeO_2_/Co–Bi showed an ∼0.5 eV negative shift compared to that of pure Co–Bi nanosheets, presumably caused by the electron transfer from CeO_2_ to Co–Bi, and it is also found that there is a more negative shift of the CeO_2_/Co–Bi hybrid with the increase in the amount of CeO_2_ (Fig. S7†). This phenomenon has been demonstrated in previous reports.^24,26,50^ Moreover, this modification in the electronic structure makes CeO_2_ more acidic (Lewis acid) and thus facilitates the activation of H_2_O molecules (Lewis base), benefiting the formation of OOH_ad_ on the surface of the composites and facilitating the OER process and thus enhancement of the efficiency of water oxidation.^24^ Additionally, the increase in the charge conductivity of CeO_2_/Co–Bi after introducing CeO_2_ indicates faster charge transfer during the catalytic reaction, improving the catalytic efficiency of water oxidation (see ESI Fig. S6†). After introducing the small CeO_2_ nanoparticles, the hybrids display a larger surface area, smaller charge transfer, better charge conductivity and stronger synergistic effects. All these advantages of the hybrids together are responsible for the good OER catalytic activity of the new CeO_2_/Co–Bi catalysts.

**Fig. 5:**
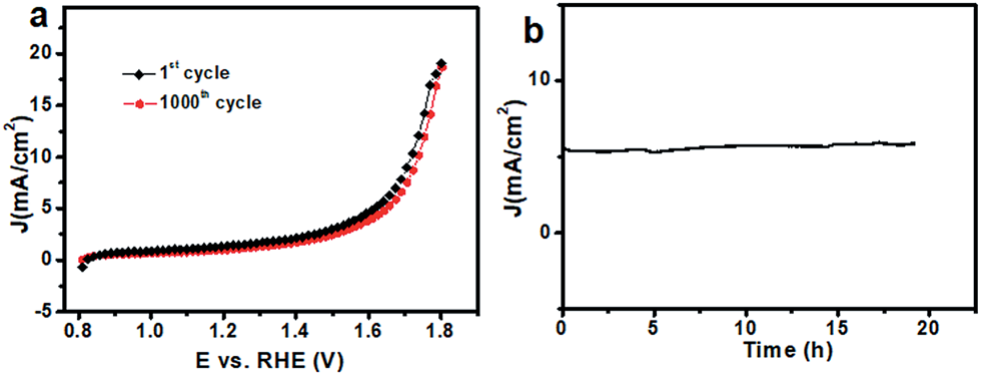
(a) Polarization curves of 20CeO_2_/Co–Bi before and after 1000 CV cycles in PBS; (b) chronoamperometric response of the as-prepared 20CeO_2_/Co–Bi composite recorded at a constant potential of 1.65 V *vs.* RHE.

Besides the catalytic activity, the stability of the catalyst, another major concern in designing cost-effective OER catalysts, was investigated as well. After 1000 CV cycles in 0.1 M PBS (Fig. S8†), there was almost no obvious loss in catalytic current (Fig. 5a), suggesting its superior stability. Additionally, the stability of the 20CeO_2_/Co–Bi composite was determined by measuring chronoamperometric responses (*i*–*t*) at 1.65 V (*vs.* RHE) in 0.1 M PBS. During the measurements, the working electrode was continuously rotating at 1600 rpm to remove the generated O_2_ bubbles. As shown in Fig. 5b, the stabilized current density indicates no obvious deactivation of the 20CeO_2_/Co–Bi composite over 19 h. The durability test of the 20CeO_2_/Co–Bi electrode under higher current density was also performed, as shown in Fig. S9.† The current density of the 20CeO_2_/Co–Bi composite over 30 h was only slightly attenuated. The results suggest that the 20CeO_2_/Co–Bi composite exhibits good catalytic stability under neutral conditions.

## 4. Conclusions

In summary, we have successfully synthesised CeO_2_/Co–Bi composites by facile chemical methods at room temperature. The larger surface area, smaller charge transfer, better charge conductivity and stronger synergistic effects obtained after introducing CeO_2_ nanoparticles are responsible for the good OER catalytic activity of these new CeO_2_/Co–Bi composite catalysts. The 20CeO_2_/Co–Bi composite exhibits outstanding OER catalytic activity with an overpotential of 453 mV at a current density of 10 mA cm^−2^, Tafel slope of around 120 mV dec^−1^ and long-term stability in neutral medium, promising for practical application in the electrolysis of water.

## Conflicts of interest

There are no conflicts to declare.

## Supplementary Material

NA-001-C9NA00356H-s001
