# Supplementary material for: Ceria/cobalt borate hybrids as efficient electrocatalysts for water oxidation under neutral conditions
Source: Nanoscale Adv. 2019 Jul 19;1(9):3686–92. doi: 10.1039/c9na00356h (PMC9417256; doi:10.1039/c9na00356h)
Supplement: NA-001-C9NA00356H-s001 [file NA-001-C9NA00356H-s001.pdf]

Electronic Supplementary Material (ESI) for Nanoscale Advances.
This journal is © The Royal Society of Chemistry 2019

Ceria/Cobalt Borate Hybrids as Efficient Electrocatalysts for Water Oxidation in Neutral Conditions

Xuemei Zhou[a], Sijia Guo[a], Qiran, Cai[b], Shaoming Huang*,[a]

[a]School of Material and Energy, Guangdong University of Technology, Guangzhou, 5 10006, China.

E-mail: smhuang@gdut.edu.cn

[b]Institute for Frontier Materials, Deakin University, Geelong Waurn Ponds Campus, Victoria, 3216, Australia.

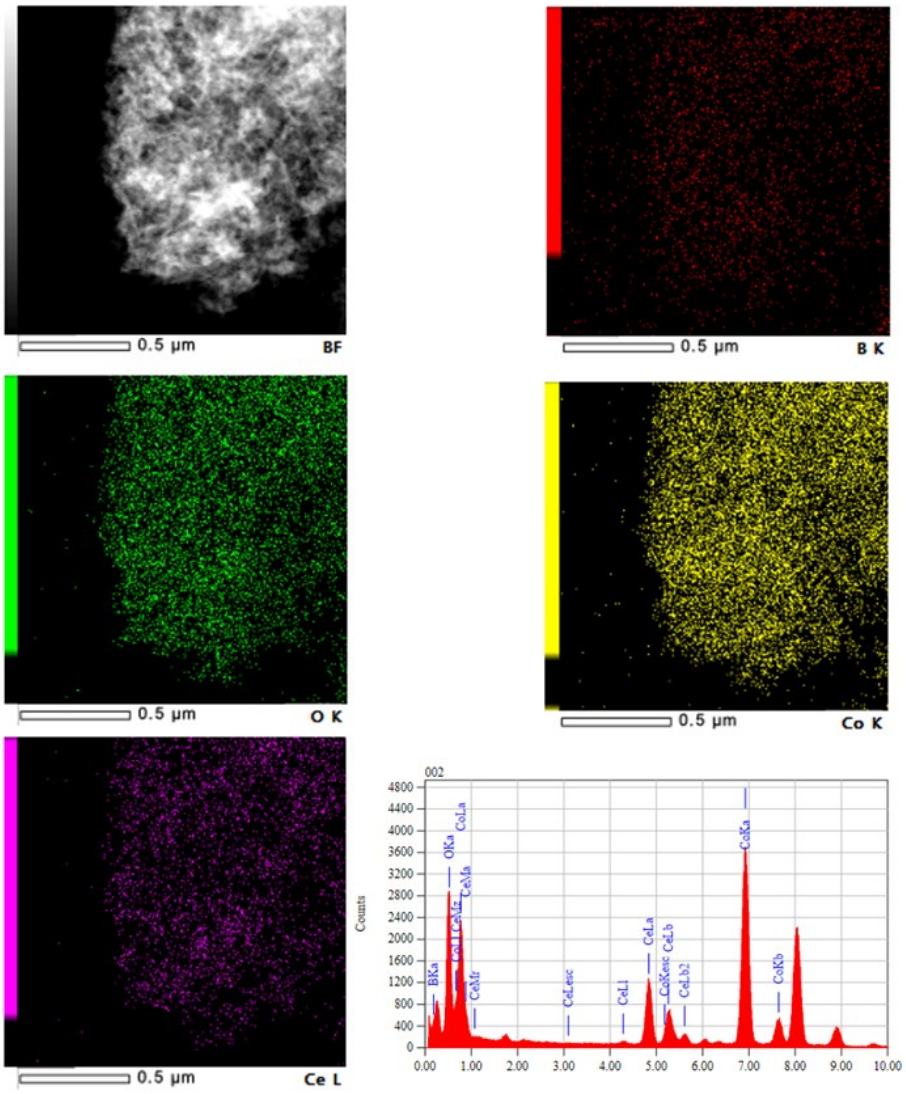

Fig. S1 EDS mapping images for $20CeO_2$/Co-Bi.

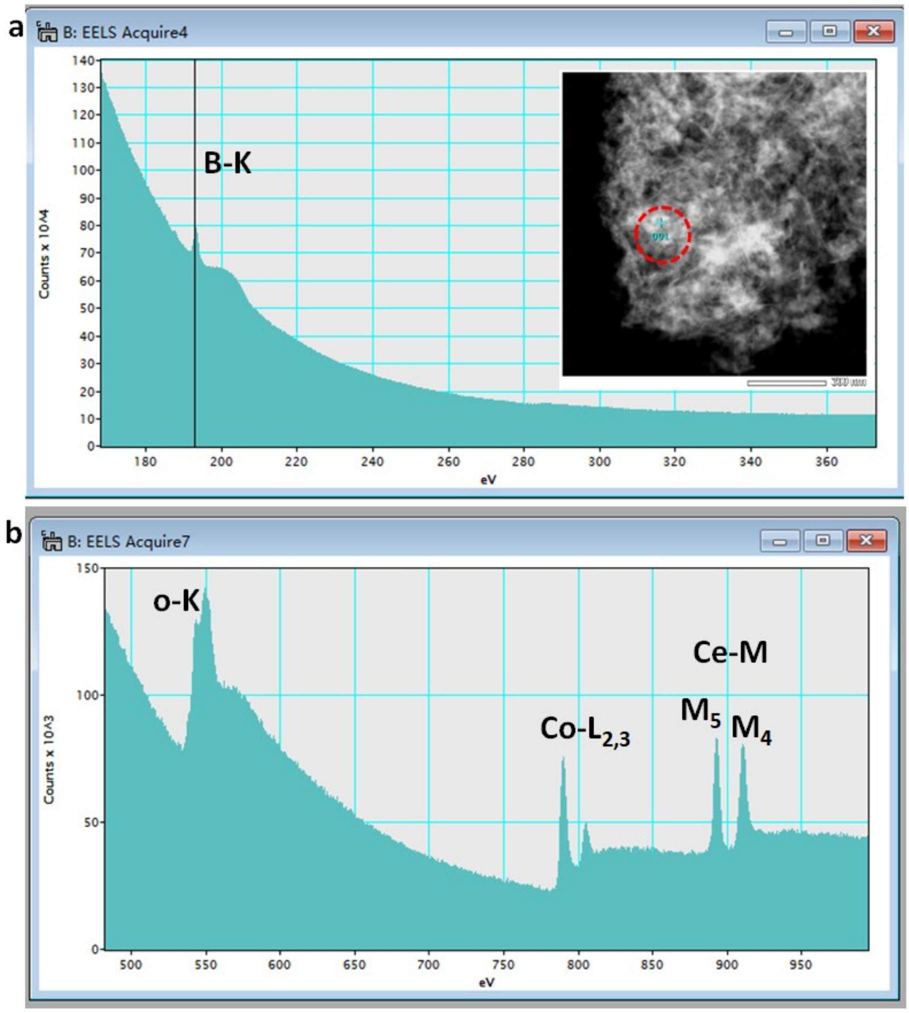

Fig. S2 EELS spectrum of $20CeO_2$/Co-Bi measured from the region of inset TEM images.

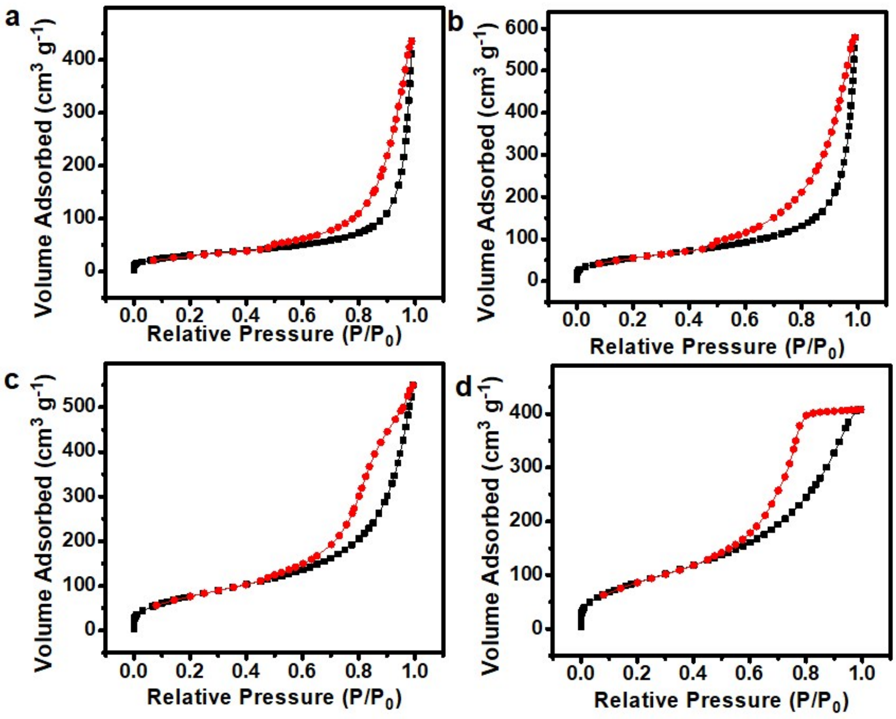

Fig. S3 Nitrogen adsorption/desorption isotherms of the Co-Bi (a),10CeO_2/Co-Bi (b), 20CeO_2/Co-Bi (c) and30CeO_2/Co-Bi (d).

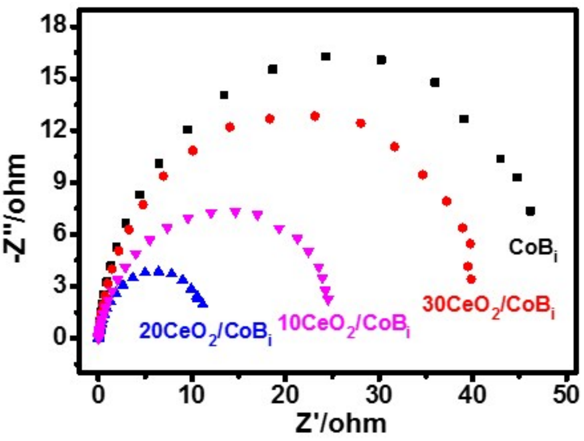

Fig. S4 Impedance Nyquist plots of Co-Bi, 10CeO_2/Co-Bi, 20CeO_2/Co-Bi and 30CeO_2/Co-Bi composites electrodes.

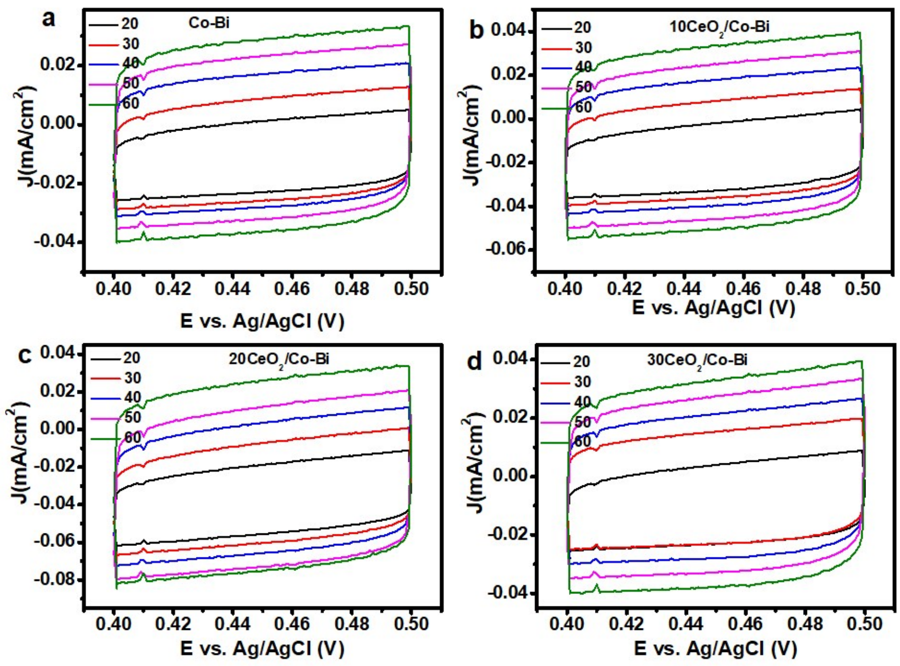

Fig. S5 Cyclic voltammograms for all the samples (Co-Bi, $10CeO_2$/Co-Bi, $20CeO_2$/Co-Bi and $30CeO_2$/Co-Bi composite) in the non-faradaic capacitance current range at scan rates of 10, 20, 30, 40, 50 and 60 mV s^{-1}.

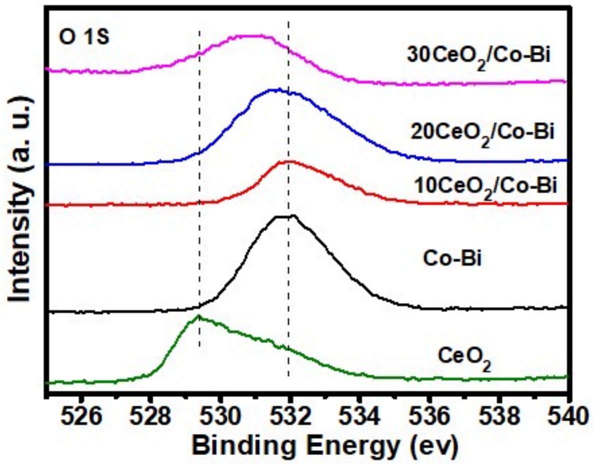

Fig. S6 High-resolution XPS spectra for O of pure CeO_2, Co-Bi, $10CeO_2$/Co-Bi, $20CeO_2$/Co-Bi and $20CeO_2$/Co-Bi hybrid.

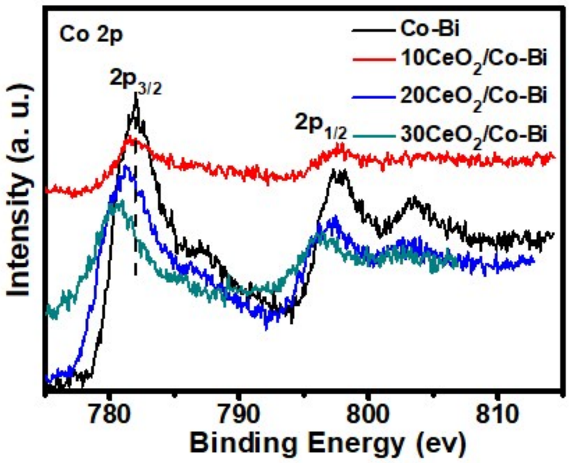

Fig. S7 High-resolution XPS spectra for Co of pure Co-Bi, $10CeO_2$/Co-Bi, $20CeO_2$/Co-Bi and $20CeO_2$/Co-Bi hybrid.

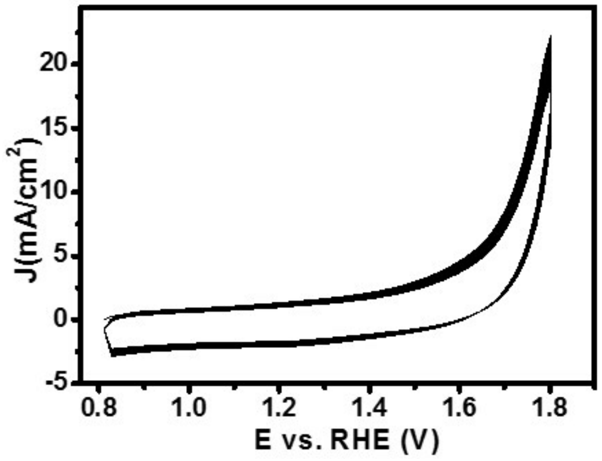

Fig. S8 Cyclic voltammograms for $20CeO_2$/Co-Bi composite electrode.

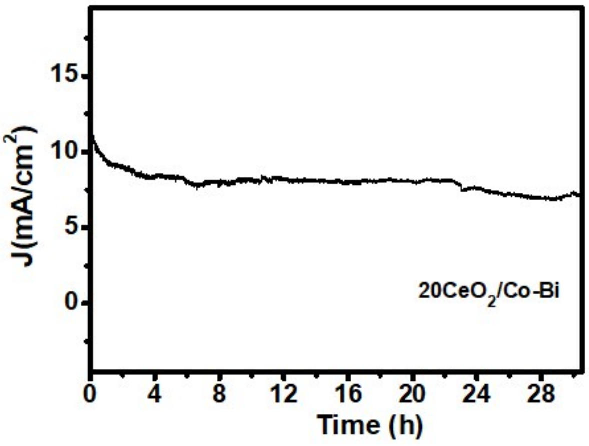

Fig. S9 Chronoamperometric response of of as-prepared $20CeO_2$/Co-Bi composite recorded at a constant potential of 1.70V vs. RHE.

Table S1 Comprision of cobalt-based catalysts of OER activities in neutral medium.

sample	Specific current density (mA cm^{-2})	Overpotential (mV)	Electrolyte concentration	Ref
Co-Pi/ITO	1	410	0.1 M KP_i	1
Co-Ni LDHs	1	490	0.1MP_i+1.73MKNO_3	2
Mn_5O_8 Nanoparticles	5	580	0.3 M PBS	3
$Co(PO_3)_2$	8	440	0.1 M phosphate	4
Co-W	1	420	0.05 M PBS	5
Sub-MnO_x	5	530	0.3 M PBS	6
Fe-based film	1	480	0.1 M PBS	7
Ultrathin Co_3S_4 nanosheets	3	620	0.1 M PBS	8
$Mn_3(PO4)_2$	0.32	680	0.5 M PBS	9
cobalt hexacyano-ferrate	1	580	0.05MKP_i+1M KNO_3	10
Au-$Co(OH)_2$	1	410	0.1 M PBS	11
$LiMnP_2O_7$	0.5	680	0.5 M PBS	12
CoP_i/GO	0.23	780	0.1 M KP_i	13
CoP_i Nanoarrays/Ti	10	450	0.1 M PBS	14
α-$Co(OH)_2$ NA/CC	10	536	1.0 M PBS	15
Fe-Co_3O_4@F-Co-Bi/CC	10	420	0.1 M K-Bi (pH=9.2)	16
Co-Bi NA/Ti	10	420	0.1 M K-Bi (pH=9.2)	17
NiB_i/CC	10	470	0.1 M KB_i (pH=9.2)	18
Co@Co-Bi/Ti	10	470	0.4 M NaBi	19
Co-Bi NS/G	10	490	0.1 M PBS	20
$20CeO_2/CoB_i$ composite	10	453	0.1 M PBS (pH=7.4)	This work

References

1 M. W. Kanan and D. G. Nocera, *Science*, 2008, **321**, 1072-1075.

2 Y. Zhang, B. Cui, C. Zhao, H. Lin and J. Li, *Phys. Chem. Chem. Phys.*, 2013, **15**, 7363-7369.

3 D. Jeong, K. Jin, S. E. Jerng, H. Seo, D. Kim, S. H. Nahm, S. H. Kim and K. T. Nam, *ACS Catal.*, 2015, **5**, 4624-4628.

4 H. S. Ahn and T. D. Tilley, *Adv. Funct. Mater.*, 2013, **23**, 227-233.

5 B. Zhang, X. Wu, F. Li, F. Yu, Y. Wang and L. Sun, *Chem. Asian J.*, 2015, **10**, 2228-2233.

6 K. Jin, A. Chu, J. Park, D. Jeong, S. E. Jerng, U. Sim, H.-Y. Jeong, C. W. Lee, Y.-S. Park and K. D. Yang, *Sci. Rep.*, 2015, **5**, 10279.

7 Y. Wu, M. Chen, Y. Han, H. Luo, X. Su, M. T. Zhang, X. Lin, J. Sun, L. Wang and L. Deng, *Angew. Chem. Int. Ed*, 2015, **54**, 4870-4875.

8 Y. Liu, C. Xiao, M. Lyu, Y. Lin, W. Cai, P. Huang, W. Tong, Y. Zou and Y. Xie, *Angew. Chem. Int. Ed*, 2015, **127**, 11383-11387.

9 K. Jin, J. Park, J. Lee, K. D. Yang, G. K. Pradhan, U. Sim, D. Jeong, H. L. Jang, S. Park and D. Kim, *J. Am. Chem. Soc.*, 2014, **136**, 7435-7443.

10 S. Pintado, S. Goberna-Ferrón, E. C. Escudero-Adán and J. R. n. Galán-Mascarós, *J. Am. Chem. Soc.*, 2013, **135**, 13270-13273.

11 Y. Zhang, B. Cui, Z. Qin, H. Lin and J. Li, *Nanoscale*, 2013, **5**, 6826-6833.

12 J. Park, H. Kim, K. Jin, B. J. Lee, Y.-S. Park, H. Kim, I. Park, K. D. Yang, H.-Y. Jeong and J. Kim, *J. Am. Chem. Soc.*, 2014, **136**, 4201-4211.

13 J. Tian, H. Li, A. M. Asiri, A. O. Al‐Youbi and X. Sun, *Small*, 2013, **9**, 2709-2714.

14 L. Xie, R. Zhang, L. Cui, D. Liu, S. Hao, Y. Ma, G. Du, A. M. Asiri and X. Sun, *Angew. Chem. Int. Ed*, 2017, **56**, 1064-1068.

15 T. Liu, L. Xie, J. Yang, R. Kong, G. Du, A. M. Asiri, X. Sun and L. Chen, *ChemElectroChem*, 2017, **4**, 1840-1845.

16 G. Zhu, R. Ge, F. Qu, G. Du, A. M. Asiri, Y. Yao and X. Sun, *J. Mater. Chem. A*, 2017, **5**, 6388-6392.

17 X. Ren, R. Ge, Y. Zhang, D. Liu, D. Wu, X. Sun, B. Du and Q. Wei, *J. Mater. Chem. A*, 2017, **5**, 7291-7294.

18 X. Ji, L. Cui, D. Liu, S. Hao, J. Liu, F. Qu, Y. Ma, G. Du, A. M. Asiri and X. Sun, *Chem. Commun.*, 2017, **53**, 3070-3073.

19 C. Xie, Y. Wang, D. Yan, L. Tao and S. Wang, *Nanoscale*, 2017, **9**, 16059-16065.

20 P. Chen, K. Xu, T. Zhou, Y. Tong, J. Wu, H. Cheng, X. Lu, H. Ding, C. Wu and Y. Xie, *Angew. Chem. Int. Ed*, 2016, **55**, 2488-2492.
